# NUP-REPORT 1.0: A Proposed Reporting and Benchmarking Framework for Non-Upright Pedestrian Detection and Pre-Crash Safety Evaluation

**DOI:** 10.3390/s26185710

**Published:** 2026-09-09

**Authors:** Nick Barua, Masahito Hitosugi

**Affiliations:** Department of Legal Medicine, Shiga University of Medical Science, Otsu 520-2192, Shiga, Japan; hitosugi@belle.shiga-med.ac.jp

**Keywords:** non-upright pedestrian, vulnerable road user, pedestrian detection, automatic emergency braking, automated driving, sensor fusion, safety evaluation

## Abstract

**Highlights:**

**What are the main findings?**

- NUP-REPORT 1.0 proposes six reporting domains and a 30-item checklist comprising 23 core items (19 universal and four component-contingent) and seven conditional items for non-upright pedestrian sensing and pre-crash evaluation.
- A purposive 20-publication feasibility audit illustrates practical use of the checklist and documents sample-specific omissions in orientation, timing, calibration, and independent-unit uncertainty reporting.

**What are the implications of the main findings?**

- The framework makes applicability, tested and untested scenario coverage, and temporal reference points explicit, improving reproducibility without converting checklist completion into a quality or safety score.
- Version 1.0 is a provisional, testable proposal that requires external consensus, inter-laboratory evaluation, and future updating before it could be treated as a validated reporting standard.

**Abstract:**

Pedestrian-detection research and pre-crash safety assessment predominantly represent upright pedestrians, although prone, supine, lateral, seated, crouched, kneeling, partially collapsed, and fall-transition states alter target geometry, visibility, sensor signatures, and intervention time. Cross-study comparison is further limited by inconsistent posture labels, data provenance, latency boundaries, uncertainty reporting, and vehicle-response assumptions. We developed NUP-REPORT 1.0 as a provisional reporting and benchmarking framework through a structured narrative synthesis of a 45-source derivation corpus covering epidemiology, sensing benchmarks, uncertainty and assurance methods, reporting-guideline methodology, and public safety protocols. A reconstructed decision ledger documented 46 candidate concepts: 30 were retained as checklist items, 10 were assigned to an extended descriptor set, and six were merged. Each retained item was mapped to supporting evidence and classified as universal core (*n* = 19), component-contingent core (*n* = 4), or conditional (*n* = 7). The framework comprises six domains, a scenario-coverage matrix, five non-overlapping event timestamps, detection-referenced stopping equations, and a 30-item checklist. A purposive feasibility audit of 20 publications, including the adjacent pedestrian-detection literature not designed specifically for non-upright evaluation, illustrated checklist use. Within this sample, target orientation and static-versus-transition state were each reported explicitly in five of 20 publications (25%); none of the 17 applicable papers reported both time-to-first-detection and detection distance, none evaluated confidence calibration, and none of the 20 reported independent-unit uncertainty intervals. Vehicle-response items were non-applicable to papers making no intervention claim. These observations are sample-specific and do not estimate field-wide reporting prevalence. NUP-REPORT is not a consensus standard, certification procedure, or safety score; it is a traceable Version 1.0 proposal for study design, retrospective audit, and stakeholder refinement.

## 1. Introduction

Road traffic injury remains a major global public-health problem, with pedestrians experiencing disproportionate risk [1]. People lying on the roadway form a small but exceptionally severe subgroup. Japanese nationwide data showed that pedestrians lying on the road represented 8.3% of pedestrian fatalities but only 0.6% of pedestrian casualties during 2012–2016 [2]. A subsequent analysis of 2452 such collisions reported that more than 80% occurred at night and identified impact speed, body-region injury, vehicle type and hit-and-run involvement as important correlates of severe or fatal outcomes [3]. These findings establish a prevention need but do not specify how perception systems should be evaluated.

Pedestrian detection has advanced through benchmark datasets, hand-crafted and learned visual representations, multispectral sensing, and multimodal driving datasets [4,5,6,7,8,9,10,11]. Large-scale driving and pedestrian-behaviour datasets have also expanded evaluation of road-scene context and crossing behaviour [11,12,13]. Yet, the dominant representation remains an upright or approximately upright person. A road-level body can occupy a wider and lower image region, present weaker conventional shape cues, overlap with debris or shadows, and produce modality-specific signatures that differ from the standing class. Consequently, similar aggregate recall values can conceal fundamentally different coverage of posture, range, illumination, occlusion, sensor state, data partitioning, and latency.

Lee et al. formalised fallen-person detection on real driving roads and released the FPD-set for direct evaluation of this corner case [14]. Recent multimodal studies have examined falling/fallen-person detection, uncertainty-aware impact reconstruction, and simulation-based pre-crash safety and injury-risk analysis [15,16,17]. Together, this work supports treating perception, timing, actuation and physical consequences as a connected chain. However, it does not provide a general reporting structure that can be applied independently of a particular dataset, sensor suite, model architecture or vehicle-response assumption.

The need for explicit reporting is reinforced by safety-critical machine-learning research. Neural-network confidence can be poorly calibrated [18], predictive uncertainty can deteriorate under distribution shift [19], out-of-distribution inputs can remain confidently misclassified [20], and multimodal systems can fail asymmetrically under adverse conditions [21]. Safety-relevant evaluation therefore requires more than mean average precision or overall accuracy: it requires traceable evidence about data provenance, condition-specific failure, calibration, timing and the link between perception output and vehicle response [22,23,24,25]. Related pedestrian-intention work reinforces the need to make temporal and behavioural context explicit [26], while uncertainty and validation studies further motivate calibration and system-level assurance [27,28,29].

Relevant road-vehicle safety standards, regulations, assessment protocols, and automation frameworks provide complementary terminology and boundary conditions for scenario, target, functional-safety, and vehicle-response reporting [30,31,32,33,34,35,36,37,38,39]. Versioned datasets, models, and evaluation artefacts can also reduce hidden technical debt [40].

NUP-REPORT 1.0 addresses this reporting gap with four contributions:

1. An operational vocabulary and six proposed reporting domains spanning target definition to vehicle-level safety interpretation;
2. An explicit five-timestamp event model and detection-referenced stopping formulation that prevent temporal and spatial double counting;
3. A traceable 30-item checklist comprising 23 core items (19 universal and four component-contingent) and seven conditional items, linked to supporting evidence and established reporting-guideline principles [41,42,43,44,45];
4. A purposive 20-publication feasibility application and worked example demonstrating how the checklist is used without producing an additive quality or safety score.

**Figure 1:**
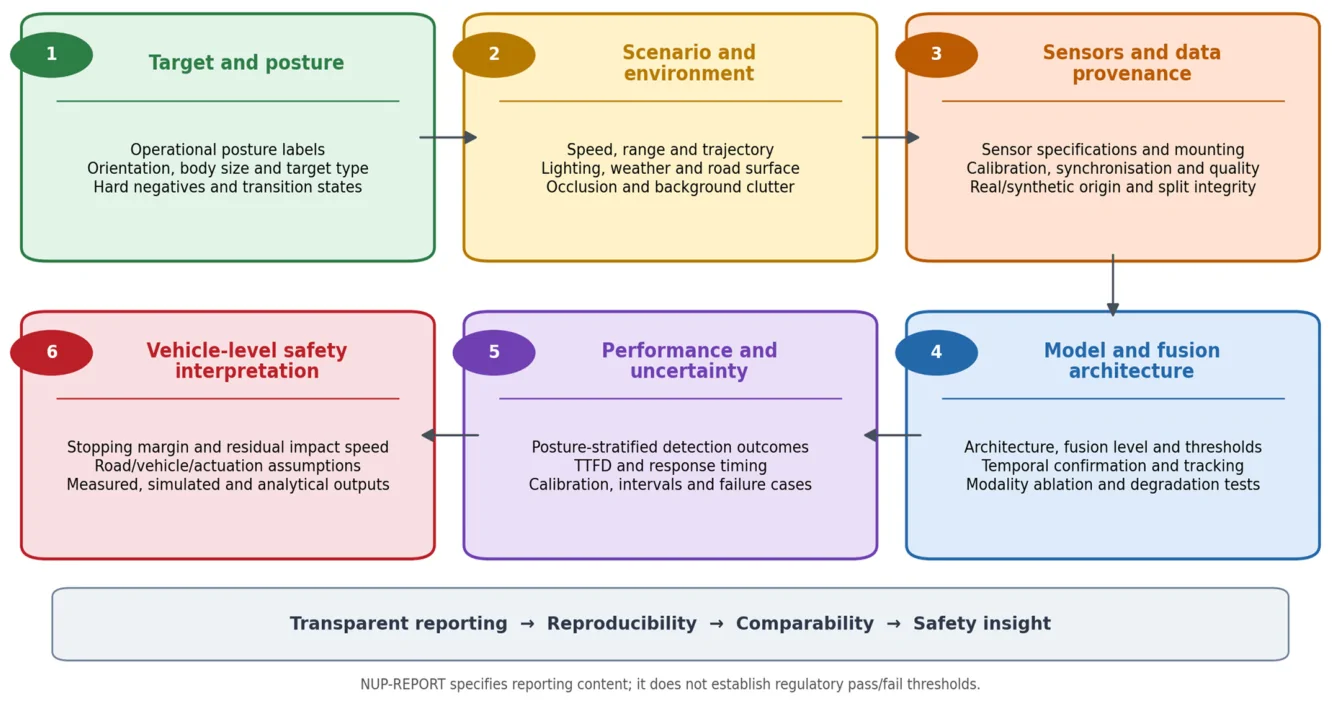
NUP-REPORT 1.0 six-domain reporting chain. The framework proceeds from target definition to vehicle-level safety interpretation and specifies reporting content rather than pass/fail thresholds.

Figure 1 summarises the six-domain reporting chain.

## 2. Scope, Terminology and Framework Development

### 2.1. Intended Use

NUP-REPORT is intended for studies of systems that detect a person in a non-upright configuration in or near a vehicle path. It can be applied to visible-spectrum cameras, thermal imaging, radar, LiDAR, ultrasonic sensing, event cameras, multimodal fusion or other relevant modalities, and to controlled-track experiments, laboratory studies, naturalistic datasets, simulation, digital twins and hybrid validation programmes.

The intended audience includes computer-vision researchers, automotive and ITS engineers, injury-prevention and forensic researchers, test laboratories, consumer-assessment organisations, regulators and standards specialists. The framework can be used prospectively during study design or retrospectively when appraising published evidence.

NUP-REPORT is not a certification or homologation standard, a replacement for Euro NCAP, UNECE, NHTSA or ISO procedures, a validated performance threshold, evidence that a detector is deployment-ready, or a claim that modelled stopping or injury outcomes represent observed real-world benefit.

### 2.2. Operational Definition of a Non-Upright Pedestrian

A non-upright pedestrian is defined here as a person whose observable body configuration differs materially from the standing, normally walking or running posture assumed in conventional pedestrian-detection datasets and vehicle test targets. The term describes posture, not cause. It should therefore not be replaced by causal labels such as “intoxicated”, “unconscious” or “medically incapacitated” unless those states are independently established.

Table 1 provides the proposed operational posture vocabulary. Studies may add subclasses, but should preserve observable configuration and orientation information needed for comparison.

### 2.3. Framework-Development Method and Evidence Synthesis

NUP-REPORT was developed as a structured narrative framework proposal, not as a systematic review or consensus guideline. The evidence update covered the period from inception to 19 August 2026. Searches were performed in PubMed/MEDLINE, Scopus, Web of Science, IEEE Xplore, Google Scholar, and official repositories maintained by ISO, Euro NCAP, UNECE, NHTSA, SAE International, WHO, and the EQUATOR Network. Search concepts combined posture terms (non-upright, prone, supine, fallen, lying, crouched, seated, kneeling, collapse), road-user and sensing terms (pedestrian, vulnerable road user, detection, camera, thermal, radar, LiDAR, multispectral, sensor fusion), and evaluation terms (latency, time-to-detection, calibration, uncertainty, stopping distance, automatic emergency braking, reporting guideline). Exact reproducible search strings, repositories, and selection rules are provided in Supplementary Section S1.

Sources were retained when they informed at least one reporting decision and represented: (i) roadway epidemiology or injury severity; (ii) pedestrian, traffic-scene, multispectral, or fallen-person benchmarks; (iii) calibration, uncertainty, distribution shift, and safety assurance; (iv) scenario-based automated-vehicle evaluation; (v) reporting-guideline development or AI-reporting guidance; or (vi) public safety standards, regulations, and assessment protocols. Indoor fall-detection studies without road-transport relevance, duplicate reports, marketing material, and sources that did not change a reporting decision were excluded. Forty-five sources were retained in the derivation corpus. Because the original exploratory search did not preserve a complete export of every search hit, we do not present a retrospective PRISMA flow or claim systematic-review completeness; this limitation is stated explicitly.

A failure-chain decomposition—target representation → sensing/data → model inference → uncertainty/timing → vehicle response → safety interpretation—generated an initial concept set. A dated reconstruction from working drafts and source notes produced 46 candidate concepts. Thirty were retained as separately auditable items, 10 context-specific descriptors were assigned to the extended set, and six overlapping concepts were merged. Retention was required because omission could impede reproduction, invalidate cross-study comparison, conceal a safety-critical subgroup failure, make timing or reference distance ambiguous, conflate measured with modelled evidence, or overstate the connection between detection and collision avoidance. The complete decision ledger and item-to-source traceability matrix appear in Supplementary Tables S2 and S3.

The resulting items were compared with TRIPOD + AI, STARD-AI, CLAIM, CONSORT-AI, and methodological guidance for developers of reporting guidelines [41,42,43,44,45]. These established guidelines informed general principles such as transparent participant/data flow, prespecification, reference standards, model and threshold reporting, uncertainty, and failure analysis. NUP-REPORT adds transport-specific elements: observable non-upright posture and orientation, road-scene scenario coverage, sensor degradation and missing-modality states, a non-overlapping event timeline, detection-referenced stopping calculations, and separation of perception from vehicle-level evidence. No Delphi panel, formal stakeholder vote, external expert panel, or inter-laboratory validation was performed; Version 1.0 is therefore a testable proposal rather than a validated standard.

Within the 23 core items, 19 are universal core items (Core-U): they are expected for every study within the intended NUP-REPORT scope and may be coded only as reported (R) or not reported (NR). Four are component-contingent core items (Core-C): they remain core requirements when the corresponding fundamental study component is present, but may be coded as not applicable (NA), with a stated reason, when that component is genuinely absent. The remaining seven items are conditional: they become applicable only when the study uses the specified optional method or makes the corresponding timing, calibration, vehicle-response, or safety-outcome claim.

Generative AI was used during manuscript preparation for language and structural editing and figure-layout assistance; it was not treated as an evidence source or autonomous reviewer. The authors verified the search record, classifications, citations, equations, figures, and conclusions and retain full responsibility.

## 3. Why Conventional Reporting Is Insufficient

### 3.1. Dataset Representation and Hidden Pooling

Pedestrian datasets differ in geographic coverage, camera placement, target scale, occlusion, annotation policy and train/test construction [4,5,6,7,8,9,10,11]. Additional pedestrian and multispectral detection studies further illustrate variation in scale, modality, cross-modal alignment, and generalisation [46,47,48,49,50,51,52]. Non-upright detection adds variation in torso orientation, projected aspect ratio, limb configuration, road-plane contact and resemblance to non-human objects. These factors make cross-study comparison unreliable when they remain implicit.

A study can also appear large while containing little independent evidence. Frame-level random splitting can place adjacent frames from the same sequence, actor, location or rendered scene in both training and testing, inflating apparent generalisation. NUP-REPORT therefore treats sequence, event, actor, location and synthetic seed as relevant units of independence.

Direct fallen-person datasets such as FPD-set provide valuable real-road test material [14], while synthetic data remain useful because rare or hazardous configurations are difficult to collect at scale. Real and synthetic samples should therefore be enumerated separately. Rendering engine, sensor model, target model, domain randomisation, noise assumptions and the role of synthetic data in training and testing should be disclosed; simulation-only results should not be described as real-world validation.

### 3.2. Pooled Accuracy Can Conceal the Safety-Critical Subgroup

Overall accuracy, precision, recall and mean average precision remain useful but can conceal poor performance in rare subgroups. A dataset dominated by upright daytime targets may yield high aggregate recall even if prone nighttime targets are frequently missed, and pooled results can hide deterioration under rain, reflective roads, occlusion, contamination or modality loss.

NUP-REPORT therefore requires posture-specific sample counts and outcomes, with additional stratification by range, illumination, occlusion and sensor state when sample size permits. Untested cells should remain visible rather than being implicitly treated as covered.

### 3.3. Confidence Is Not Equivalent to Reliability

A detector confidence score is not automatically a calibrated probability of correctness. When confidence controls fusion, temporal confirmation, warning or braking, calibration should be reported using appropriate measures such as expected calibration error (ECE), reliability diagrams or Brier score [18] and should be re-evaluated under distribution shift [19].

Out-of-distribution detection, selective prediction and abstention can be useful, but their false-negative and latency consequences must also be reported. A system that abstains correctly in a benchmark can still leave insufficient time for physical intervention.

### 3.4. Inference Time Is Not Total Response Latency

Inference time alone is not total observation-to-brake response time. NUP-REPORT therefore uses five observable event timestamps: t_obs_, first eligible sensor observation; t_det_, first detector output satisfying the prespecified criterion; t_dec_, intervention decision available; t_cmd_, actuator command issued; and t_brake_, effective brake-force onset. Every component is a difference between adjacent timestamps, so no interval can be counted twice.

The corresponding intervals are:

- TTFD = t_det_ − t_obs_: sensing, readout, preprocessing, inference, and any detector-side criterion needed to produce the first valid output;
- Decision-logic delay = t_dec_ − t_det_: tracking, persistence, fusion, or decision logic after the first criterion-satisfying output;
- Command-transfer delay = t_cmd_ − t_dec_: transmission of the available decision to the actuator interface;
- Actuation/build-up delay = t_brake_ − t_cmd_: actuator response until effective brake force begins;
- Post-detection delay = t_brake_ − t_det_; total observation-to-brake delay = t_brake_ − t_obs_.

**Figure 2:**
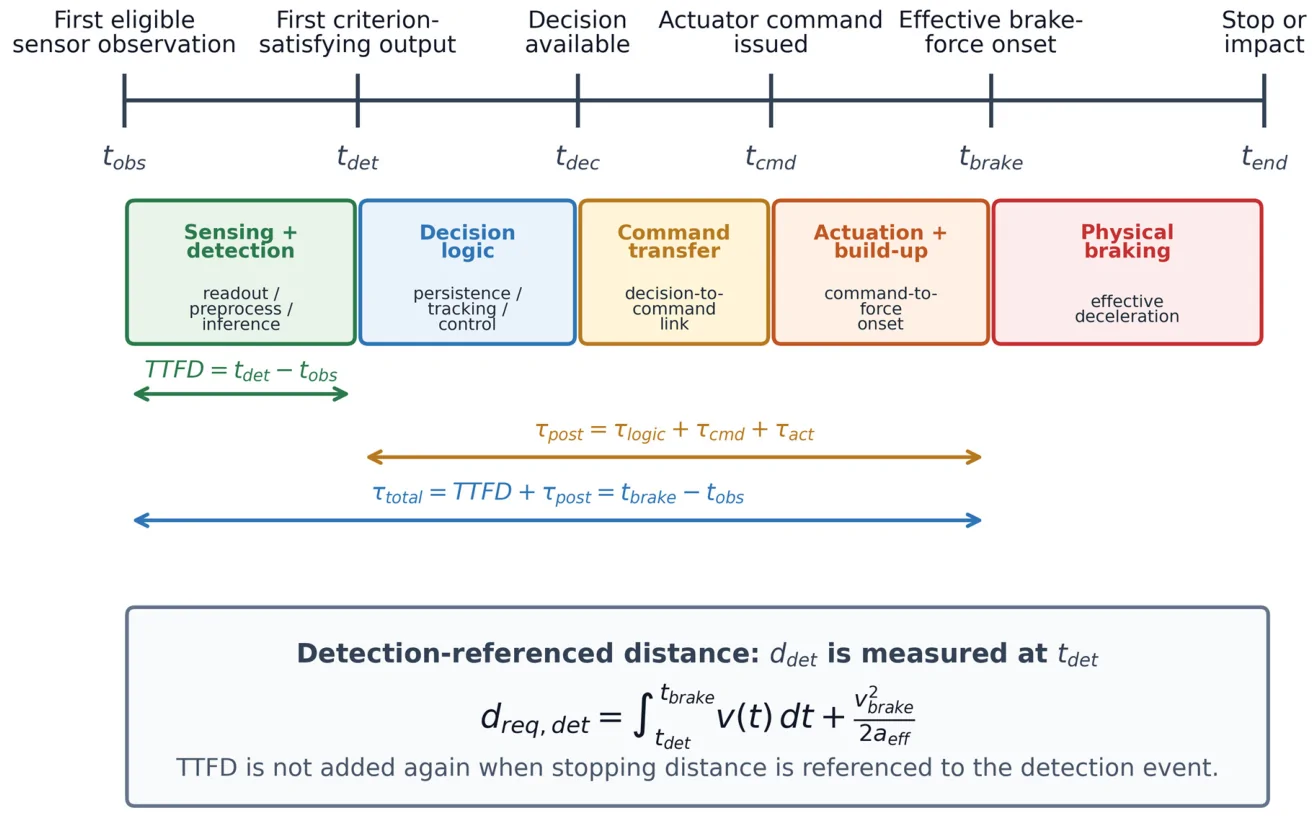
Unambiguous event timeline and detection-referenced stopping formulation. Adjacent timestamp differences define non-overlapping latency components. TTFD ends at the first criterion-satisfying output; stopping distance measured at that event uses only the subsequent trajectory.

Figure 2 fixes the temporal and spatial reference points. If detection distance is measured at t_det_, the required stopping distance includes only the post-detection trajectory from t_det_ onward; TTFD is not added again.

## 4. The Six NUP-REPORT Domains

As defined in Section 2.3, NUP-REPORT distinguishes universal core (Core-U), component-contingent core (Core-C), and conditional items. Table 2 summarises the six reporting domains and the corresponding checklist applicability categories.

### 4.1. Domain 1: Target and Posture

Every study should define what counts as a positive target. Because prone people, bags, blankets, road debris and shadows can have similar two-dimensional geometry, negative sets should include difficult non-human objects with road-level signatures. Human participants, mannequins, articulated targets, digital humans and simplified synthetic objects should be reported separately, together with the properties that are and are not human-representative.

For fall or collapse sequences, frame-level labels should distinguish transition from the final stable posture, and any prediction claim should define the event boundary and lead time prospectively.

### 4.2. Domain 2: Scenario and Environment

Scenario variables determine both detectability and physical intervention opportunity. Illumination/headlamp state and occlusion/background clutter are universal core items. Ego speed and target motion state (Item 7), initial range and lane position (Item 8), and weather/road-surface condition (Item 10) are component-contingent core items: they are required when the study captures, simulates, or claims the corresponding dynamic, geometric, or environmental component and otherwise may be marked NA with a reason. Illumination and weather severity should be quantified when practicable, with glare and sensor contamination reported when relevant.

Detection distance should use a defined target reference point. When lateral offset or road curvature materially affects line of sight, the coordinate convention and relevant geometry should also be stated.

### 4.3. Domain 3: Sensors and Data Provenance

Sensor reporting should allow another laboratory to reconstruct the physical measurement process: modality, resolution or angular resolution, field of view, frame/scan rate, and mounting position should be stated. Calibration and synchronisation (Item 13) are component-contingent cores: they are required whenever such procedures are performed, needed by the sensing configuration, or available from the dataset, and may be coded as NA only when no corresponding procedure exists in the study design—not when it is merely unreported. Data provenance should identify source, collection period, geographic context, annotation procedure and the unit of independence; real, augmented and synthetic data should be separately enumerated.

Sequence-level leakage requires particular attention. Adjacent frames, repeated actors, near-identical simulations or the same location under minor variation should not be randomly split across training and testing without justification or sensitivity analysis. Group-wise partitioning by event, actor, location and synthetic seed is preferred.

### 4.4. Domain 4: Model and Fusion Architecture

Studies should report model family/version, input resolution, pretraining and fine-tuning, class definitions, confidence and matching thresholds, tracking/temporal confirmation, fusion level, compute hardware and deployed numerical precision. Warm-up, data transfer and pre/post-processing should be included in latency measurements or explicitly excluded.

Multimodal studies should include modality-specific baselines and controlled ablation or degradation tests. Missing-modality handling—such as zero filling, masking, fallback models, confidence reweighting or system shutdown—should be disclosed because these strategies are not safety-equivalent.

### 4.5. Domain 5: Performance and Uncertainty

Performance reporting should include sample size, precision, recall, false-negative count and an uncertainty interval for each principal posture class. Object-detection studies should specify the intersection-over-union and matching policy; sequence-based studies should define the valid-detection persistence criterion. Both frame-level outcomes and event-level safety-critical misses should be reported, with denominators explicit for each subgroup.

When confidence affects fusion or intervention, calibration should be evaluated overall and under relevant shifts. Failure analysis should include representative false positives and false negatives, especially near-threshold and high-confidence failures, with modality-specific evidence when available.

### 4.6. Domain 6: Vehicle-Level Safety Interpretation

A detection result becomes safety-relevant only through time, distance and vehicle response. Studies conducting temporal/system evaluation should report TTFD and detection distance at the first valid output (Item 23); studies making intervention claims should additionally report post-detection and total observation-to-brake timing (Item 24). Vehicle-response assumptions (Item 28) and stopping-margin or residual-speed outcomes (Item 30) are conditional on vehicle-response modelling and vehicle-safety claims, respectively. Measured vehicle tests, simulation and analytical calculations should always be labelled separately (Core-U Item 29), and uncertainty in dominant assumptions should be propagated or explored by sensitivity analysis where feasible.

Modelled residual impact speed or injury probability should not be described as observed effectiveness. Sensitivity analysis should vary assumptions that materially influence the result, particularly detection distance, response delay and effective deceleration.

## 5. Proposed Benchmark Scenario Matrix

The proposed scenario matrix is a coverage-reporting device, not a mandatory fully factorial experiment. Studies should display tested, untested, and non-applicable cells and explain how combinations were prioritised. Table 3 lists candidate dimensions; exact levels should be justified for the operational design domain.

**Figure 3:**
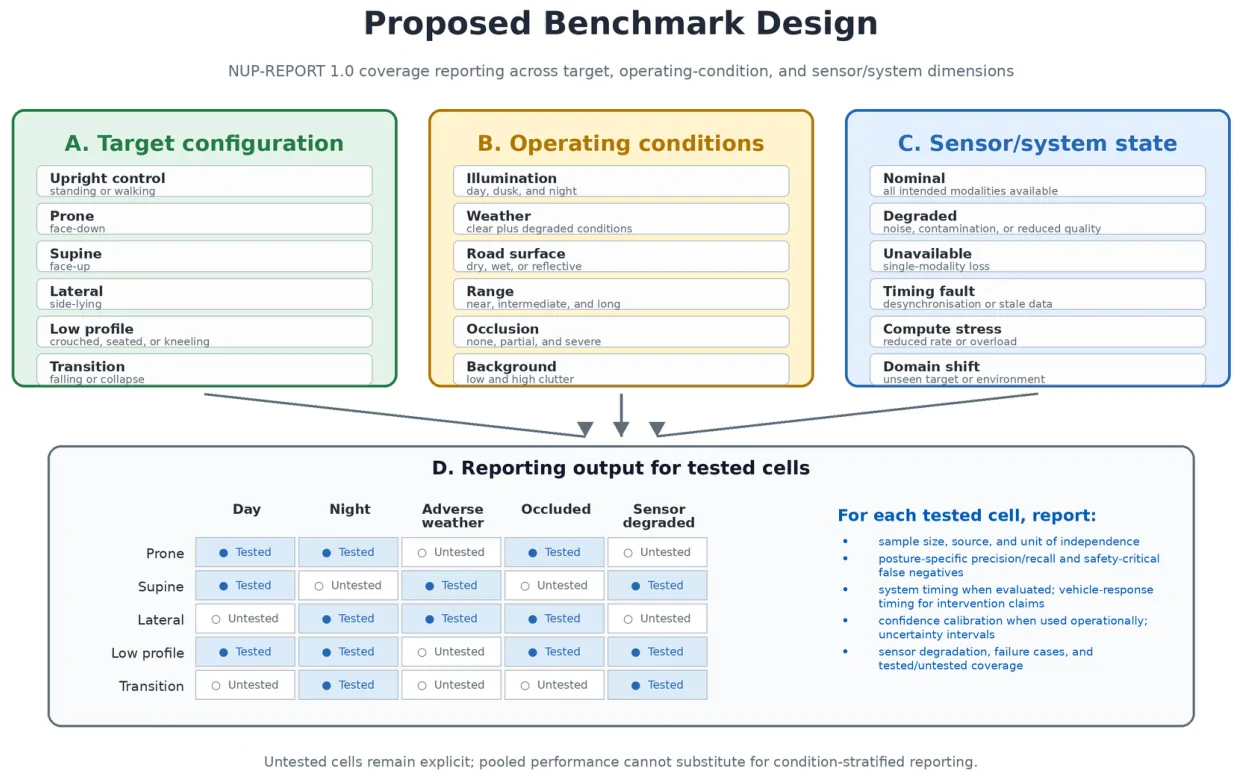
Proposed benchmark design. Target, operating-condition, and sensor/system-state dimensions converge on an explicit coverage map. Filled and open markers indicate illustrative tested and untested cells only; they do not represent performance results. The reporting-output panel distinguishes universal results from timing, calibration, and vehicle-response information that is required only when the corresponding evaluation or claim is present.

Figure 3 visualises how target configuration, operating conditions, and sensor/system state converge on an explicit coverage map. The matrix is illustrative: its purpose is to require transparent reporting of tested and untested scenario cells rather than to encode performance values.

## 6. Proposed Outcome Definitions and Equations

### 6.1. Time-to-First-Detection

Let t_obs_ denote the first eligible sensor observation and t_det_ the first detector output satisfying the prespecified confidence, matching, and detector-side persistence criterion. If the intervention system requires additional tracking or confirmation after that output, the additional interval begins at t_det_ and belongs to decision-logic delay:

$$TTFD=t_{det}-t_{obs}$$

Eligibility should be defined prospectively from the field of view, visible target area, range, or a known simulation onset rather than selected after inspecting outputs. Inference time may be profiled separately, but it remains nested within TTFD whenever t_det_ is defined as the first available criterion-satisfying detector output.

### 6.2. Post-Detection and Total Response Delay

The remaining timestamps make post-detection processing explicit. Let t_dec_ be decision availability, t_cmd_ the actuator command, and t_brake_ the effective brake-force onset. The adjacent intervals and their sums are:

$$\tau_{logic}=t_{dec}-t_{det},\;\tau_{cmd}=t_{cmd}-t_{dec},\;\tau_{act}=t_{brake}-t_{cmd}\;\tau_{post}=\tau_{logic}+\tau_{cmd}+\tau_{act}=t_{brake}-t_{det}\;\tau_{total}=TTFD+\tau_{post}=t_{brake}-t_{obs}$$

The adjacent intervals are mutually exclusive. The post-detection sum is the only processing interval used after d_det_ has been measured at t_det_. TTFD is used only when reporting the complete observation-to-brake chain from t_obs_.

### 6.3. Time to Collision

For a stationary target and constant positive longitudinal relative speed *v_rel_*:

$$TTC=\frac{d}{v_{rel}}$$

When relative speed, curvature or target motion varies materially, the study should state and justify the alternative TTC model used.

### 6.4. Required Stopping Distance from First Valid Detection

Let d_det_ be the longitudinal distance available at t_det_. The distance required from that detection event is the distance travelled until effective brake-force onset plus the braking distance thereafter. With a stationary target, constant effective deceleration a_eff_ > 0, and approximately constant pre-brake speed, the general expression and its common approximation are:

$$d_{req,det}=\int_{t_{det}}^{t_{brake}}v\left(t\right)\;dt+\frac{v_{brake}^{2}}{2a_{eff}}\approx v_{det}\tau_{post}+\frac{v_{det}^{2}}{2a_{eff}}$$

The integral contains only motion after first valid detection. The approximation uses v_det_ across the post-detection interval and for braking; higher-fidelity studies should use v_brake_ and the measured speed trajectory. TTFD must not be added because d_det_ is already referenced to t_det_.

### 6.5. Stopping Margin

The stopping margin is defined as the detection-referenced available distance minus the required stopping distance:

$$M_{stop}=d_{det}-d_{req,det}$$

Within this simplified longitudinal model, a positive stopping margin indicates physical stopping feasibility under the stated assumptions; it does not prove collision avoidance because steering, friction, target motion, controller constraints and surrounding traffic can alter the outcome. For moving targets, studies should use a consistent relative-motion formulation rather than the stationary-target simplification.

### 6.6. Residual Impact Speed

For the stationary-target simplification, first define the distance remaining for effective deceleration after the post-detection response delay. The corresponding idealised residual impact speed is:

$$d_{br}=max\left(0,d_{det}-\int_{t_{det}}^{t_{brake}}v\left(t\right)\;dt\right)\;v_{imp}^{2}=max\left(0,v_{brake}^{2}-2a_{eff}d_{br}\right)$$

Let *t_imp_* denote the time at which the vehicle reaches the target. Two cases must be distinguished. If *t_imp_* ≤ *t_brake_*, the available distance is exhausted before effective braking begins; impact occurs during the response interval and residual impact speed is taken from the pre-brake speed trajectory, *v_imp_* = *v*(*t_imp_*) (approximately *v_det_* under the constant pre-brake-speed assumption). Only when *t_brake_* < *t_imp_* does effective braking precede impact, in which case *d_br_* > 0 and the braking-based expression above applies. Thus, *d_br_* = 0 is a case indicator for pre-brake impact, not a licence to substitute a hypothetical *v_brake_* into the braking equation.

For the braking-before-impact case, these expressions are valid only when distance is referenced consistently to *t_det_* and constant effective deceleration is a reasonable approximation. If target motion, steering or time-varying deceleration is material, the study should report the higher-fidelity model used.

### 6.7. Expected Calibration Error

For K confidence bins *B_k_* with empirical accuracy acc(*B_k_*) and mean confidence conf(*B_k_*):

$$ECE=\sum_{k=1}^{K}\frac{\left|B_{k}\right|}{n}\left|acc\left(B_{k}\right)-conf\left(B_{k}\right)\right|$$

ECE should be accompanied by binning rules and sample counts, preferably with a reliability diagram. Adaptive or class-conditional alternatives may be appropriate for strongly imbalanced samples.

### 6.8. Safety-Critical False-Negative Rate

Let *I_i_* = 1 when event i is not validly detected before a predefined intervention boundary and *I_i_* = 0 when otherwise. For N eligible events:

$${FNR}_{SC}=\frac{1}{N}\sum_{i=1}^{N}I_{i}$$

The intervention boundary may be defined by TTC, stopping distance or a validated control criterion, but must be specified before outcome analysis. Table 4 consolidates the proposed outcome definitions.

## 7. Analysis, Uncertainty and Failure Handling

Frame-level, sequence-level and event-level analyses should be distinguished. Frame-level confidence intervals are inappropriate when thousands of correlated frames arise from a few independent events; safety claims should normally use the independent event or scenario replicate as the unit of analysis. When bootstrap intervals are used, resampling should occur at the highest relevant clustering level, such as event, actor or route, with the number of independent clusters and replicates reported.

Confidence and persistence thresholds should be selected using training or validation data rather than the final test set. Threshold sensitivity should be shown when conclusions vary materially across plausible settings; an overall F1-optimal threshold is not necessarily appropriate for a rare safety-critical class.

Missing or failed sensor data should be classified as intentionally removed, technically unavailable, corrupted, outside range or rejected by quality control. Missing values should not be silently replaced by nominal data, and the fusion or fallback strategy should be evaluated under the stated missing-modality condition.

Principal outcomes should include uncertainty intervals rather than point estimates alone. Exact binomial, Wilson or cluster-bootstrap intervals may be used as appropriate. When many subgroup comparisons are subjected to formal hypothesis testing, multiplicity should be addressed; effect sizes, uncertainty intervals and absolute error counts are generally more informative than isolated *p*-values. Versioned datasets, models and evaluation artefacts can also reduce hidden technical debt [40].

## 8. Relationship to Established Reporting Guidelines and Safety Frameworks

### 8.1. Relationship to AI Reporting Guidelines

TRIPOD + AI, STARD-AI, CLAIM, and CONSORT-AI address transparent reporting of prediction models, diagnostic-accuracy studies, medical imaging AI, and AI interventions, respectively [41,42,43,44]. NUP-REPORT adopts their general emphasis on prespecification, data provenance, reference definitions, model and threshold transparency, uncertainty, and failure analysis, while narrowing the application to non-upright road users and extending the reporting chain into sensing conditions, temporal availability, and vehicle-level physical feasibility. Table 5 identifies overlap and genuinely transport-specific additions; it does not imply formal endorsement by the guideline developers.

### 8.2. Relationship to Road-Vehicle Safety Frameworks

NUP-REPORT is intended to organise evidence that may later inform assurance or protocol development; it does not establish compliance. The framework is complementary to ISO 26262 functional safety, ISO 21448 SOTIF, ISO 34501/34502 scenario terminology and evaluation, ISO 19206-2 pedestrian targets, Euro NCAP VRU assessment, UN Regulation No. 152, FMVSS No. 127 and SAE J3016 [30,31,32,33,34,35,36,37,38,39]. The crosswalk in Table S2 of the Supplementary Materials is therefore a scope-and-boundary mapping, not a claim of certification, equivalence or endorsement.

A missing camera frame, corrupted stream or failed communication link can represent malfunctioning behaviour relevant to functional safety. By contrast, a correctly functioning perception stack that fails to recognise a prone person outside its training distribution can represent a performance insufficiency relevant to SOTIF. NUP-REPORT requires these categories to remain distinguishable rather than being pooled as generic errors.

Public vehicle-test programmes use defined, reproducible targets and scenarios. Exploratory non-upright simulations should therefore not be presented as equivalent to regulatory or consumer testing. The appropriate progression is transparent reporting, representative data, target development, inter-laboratory reproducibility, threshold research and formal stakeholder review.

## 9. Pilot Application and Worked Example

The pilot application used the Core-U, Core-C, and conditional applicability rules defined in Section 2.3. A study component was coded as not applicable only when the corresponding prespecified applicability trigger was genuinely absent.

**Figure 4:**
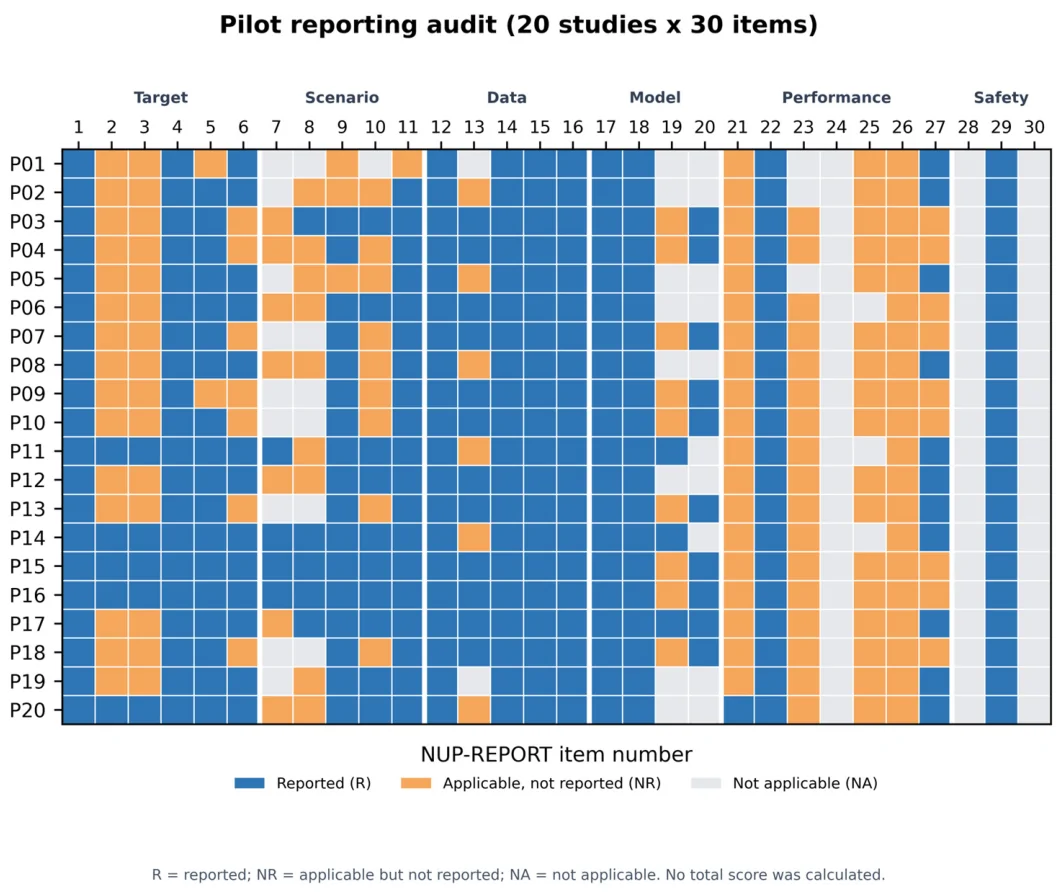
Pilot application of the 30-item NUP-REPORT checklist to 20 external publications. Blue, orange, and grey denote reported (R), not reported (NR), and not applicable (NA). Paper identifiers and full coding are provided in Supplementary Tables S6–S8. The matrix is a feasibility demonstration, describes this purposive sample only, and must not be summed as a safety or quality score or interpreted as a field-wide prevalence estimate.

Within this purposive sample, all papers defined the basic target, sensor/data source, annotation approach, and model or benchmark context. In contrast, orientation and static-versus-transition status were explicit in 5/20 papers each (25%). No applicable paper reported both TTFD and detection distance (0/17), none evaluated confidence calibration (0/17), and none provided uncertainty intervals at the independent event/sequence unit (0/20). Vehicle-response assumptions, braking delay, and residual-speed items were NA for papers making no vehicle-intervention claim, illustrating why applicability coding is preferable to treating every blank item as noncompliance. These frequencies describe the 20-paper sample only and should not be interpreted as field-wide prevalence estimates. Figure 4 visualises the item-level R/NR/NA pattern across the 20-publication sample.

### Worked Example

Consider a hypothetical thermal-camera study of a stationary, prone pedestrian at night. The detector is evaluated at two ranges and one ego speed, but the study makes no braking claim. Table 6 shows the intended logic: report posture, orientation, lighting, target and sensor details, independent test events, TTFD, detection distance, and uncertainty, and mark fusion ablation, actuator assumptions, and stopping outcomes as NA with reasons. The example exposes scenario and timing gaps without penalising a study for outcomes outside its stated scope.

## 10. Implementation Pathway and Versioning

Version 1.0 should be treated as a starting point. A credible adoption pathway progresses from framework release to dataset audit, open benchmark development, inter-laboratory study, threshold research and, only after sufficient evidence accumulates, standards engagement. Checklist completion should be reported item by item, with non-applicable items identified and justified; the number of completed items should not be converted into a safety or quality score. The staged pathway is illustrated as Figure S1 in the Supplementary Materials.

Future releases should use semantic versioning: 1.x for clarifications or added reporting items that preserve the conceptual structure; 2.0 for major restructuring after external consultation or validation; and optional domain extensions for specialised contexts such as heavy vehicles, low-speed manoeuvring, vulnerable children or wheelchair users. Each release should include a change log and an archived checklist.

## 11. Limitations

NUP-REPORT 1.0 has important limitations. First, the evidence synthesis was structured and traceable but not systematic: a complete export of all original search hits was unavailable, no formal risk-of-bias assessment was conducted, and one author performed the pilot-audit coding. The purposive sample of 20 publications demonstrates feasibility but cannot estimate the prevalence of reporting practices across the wider field. Several sampled publications were included as the adjacent pedestrian-detection or benchmark literature and were not designed specifically for non-upright pedestrian evaluation. Independent duplicate coding, agreement analysis, broader systematic sampling, stakeholder interviews, and a Delphi or comparable consensus process are required before the checklist can be treated as validated guidance.

Second, the proposed scenario matrix is intentionally broad and does not define universal parameter values. Speeds, ranges, illumination levels, target fidelity, and degradation severity depend on the operational design domain, vehicle class, sensor configuration, and intended claim.

Third, the stopping equations are deliberately simplified. Real braking and steering depend on friction, brake build-up, anti-lock control, gradient, load, curvature, target motion and surrounding traffic; higher-fidelity models should be used when these factors are material.

Fourth, reporting quality cannot compensate for unrepresentative datasets. Rare non-upright events remain difficult to capture ethically and at scale, and synthetic data or surrogates will continue to be necessary; their domain gap and fidelity limitations should be quantified. Finally, prior work by the authors is cited because it directly motivates the detection, uncertainty and safety-integration problem [15,16,17]. NUP-REPORT does not reuse those datasets, case material or unpublished material; it proposes an independent reporting structure grounded in a broader evidence base that includes independent fallen-person research [14], the benchmark literature and public standards. These limitations are reasons for staged validation, not grounds for treating Version 1.0 as a validated protocol.

## 12. Conclusions

Non-upright pedestrians present a distinct perception and pre-crash safety-evaluation problem. Cross-study comparison is unreliable when posture labels, target surrogates, data partitions, environmental conditions, sensor states, latency boundaries and vehicle-response assumptions remain implicit.

NUP-REPORT 1.0 makes these elements explicit through six domains, a proposed scenario matrix, a proposed outcome set, an unambiguous five-timestamp event model, detection-referenced stopping equations, and a traceable 30-item checklist comprising 19 universal core, four component-contingent core, and seven conditional items. Its central principle is that detection accuracy is not equivalent to safety performance.

Within the purposive 20-publication sample, the framework distinguished absent reporting from genuinely inapplicable content and identified sample-specific omissions without producing an unjustified total score. These observations are not estimates of the prevalence of reporting practices across the wider field. NUP-REPORT remains a provisional Version 1.0 proposal; external consensus, inter-rater testing, broader evidence audits, and inter-laboratory studies are the necessary next steps.

## Figures and Tables

**Table 1:** Operational posture vocabulary for NUP-REPORT.

Category	Proposed Operational Description	Recommended Additional Descriptors
Upright control	Standing, walking or running with a predominantly vertical torso	Gait phase, crossing direction, adult/child target
Prone	Torso predominantly horizontal, face or anterior surface toward the road	Head orientation, limb spread, longitudinal/transverse/oblique alignment
Supine	Torso predominantly horizontal, face or anterior surface away from the road	Head orientation, limb spread, alignment
Lateral	Side-lying or substantially rotated from prone/supine	Left/right side, curled/extended posture
Crouched/squatting	Low centre of mass with flexed hips and knees	Stationary/moving, torso angle
Seated/kneeling	Floor-level seated or kneeling configuration	Upright torso versus collapsed torso
Partially collapsed	Intermediate or asymmetric posture not represented by a stable class	Support point, body-region visibility
Transition/fall	Time-varying sequence from the upright or mobile state into a non-upright state	Transition onset, fall direction, frame-level posture labels

**Table 2:** Six NUP-REPORT domains and checklist applicability categories.

Domain	Core Checklist Items (Core-U; Core-C Where Indicated)	Conditional Checklist Items and Extended Descriptors (When Applicable)
1. Target and posture	Core-U 1–6: posture definition; orientation; static/transition state; target type; body properties; difficult negatives	No conditional checklist item. Extended: limb/head orientation; thermal properties; clothing; surrogate fidelity
2. Scenario and environment	Core-U 9, 11: illumination/headlamps; occlusion/clutter. Core-C 7, 8, 10: speed/motion; range/lane; weather/road surface	No conditional checklist item. Extended: curvature/gradient; glare; precipitation intensity; road temperature; traffic context
3. Sensors and data provenance	Core-U 12, 14–16: sensor specifications/mounting; sample provenance; annotation; independent partitioning. Core-C 13: calibration/synchronisation	No conditional checklist item. Extended: compression; dropped frames; contamination; inter-annotator agreement; release identifiers
4. Model and fusion architecture	Core-U 17–18: architecture/version/input/pretraining; confidence, matching, and persistence thresholds	Conditional 19–20: fusion/temporal or missing-modality methods; modality baselines/ablation. Extended: parameter count; precision; threshold sensitivity; compute-load tests
5. Performance and uncertainty	Core-U 21–22, 26–27: counts and posture-specific metrics; false negatives; independent-unit uncertainty intervals; representative failures	Conditional 23–25: TTFD/detection distance; intervention timing; operational confidence calibration. Extended: OOD/selective prediction; bootstrap; degradation analyses
6. Vehicle-level safety interpretation	Core-U 29: measured, simulated, or analytical status	Conditional 28, 30: vehicle-response assumptions; stopping margin/residual speed and sensitivity. Extended: steering; surrounding traffic; uncertainty propagation; injury-model provenance

**Table 3:** Proposed benchmark dimensions and candidate levels.

Dimension	Candidate Levels	Rationale
Posture	Upright control; prone; supine; lateral; crouched/seated/kneeling; transition where relevant	Separates the non-upright effect from the general detector quality
Orientation	Longitudinal; transverse; oblique	Changes projected geometry and visible body regions
State	Stationary; moving; falling/collapsing where relevant	Distinguishes static detection from temporal anticipation
Illumination	Daylight, low light, darkness with headlamps	Addresses a major epidemiological and sensing condition
Weather	Clear plus at least one degraded condition	Tests modality asymmetry and domain shift
Road surface	Dry plus wet/reflective where feasible	Affects visible, NIR, thermal and LiDAR response
Range	Near; intermediate; long	Enables distance-dependent performance and stopping-margin analysis
Occlusion	None; partial; severe	Tests low-profile visibility and context dependence
Target type	Adult-size; small-stature; at least one hard negative class	Addresses scale and false-activation risk
Sensor state	Nominal; one degraded or unavailable modality; timing fault where relevant	Evaluates graceful degradation and fusion robustness
Ego speed	Low urban plus higher urban/peri-urban speed	Connects detection timing to physical intervention opportunity
Background	Low clutter; high clutter	Tests confusion with roadside or road-surface objects

**Table 4:** NUP-REPORT outcomes and proposed reporting forms.

Outcome	Unit	Proposed Reporting Form	Safety Interpretation
Posture-specific recall	%	Estimate, numerator/denominator, confidence interval	Reveals subgroup misses hidden by pooled recall
Safety-critical FNR	% of events	Boundary definition, event count, interval	Measures failures that persist beyond intervention opportunity
TTFD	ms or s	Median, distribution and tail percentile	Captures how quickly a valid detector output becomes available
Distance to first valid detection	m	Distribution by posture and condition	Connects detection to the available stopping distance
Response timing	ms or s	TTFD, post-detection delay, total observation-to-brake delay, component decomposition and hardware	Prevents inference-only and double-counted timing claims
Detection persistence	% frames or duration	Persistence rule and distribution	Distinguishes isolated detections from stable tracking
Calibration	ECE/Brier/reliability	Overall and shifted-condition results	Assesses whether confidence supports safe weighting or decisions
Degradation sensitivity	Absolute/relative change	Modality and degradation severity	Evaluates graceful degradation and fusion dependence
Stopping margin	m	Distribution with uncertainty propagation	Indicates physical feasibility under stated assumptions
Residual impact speed	km/h or m/s	Distribution and assumptions	Estimates mitigation when stopping is not feasible

**Table 5:** Comparison with established AI reporting guidelines.

Guideline	Primary Scope	Shared Principles Used by NUP-REPORT	NUP-REPORT-Specific Additions
TRIPOD + AI	Prediction-model studies	Data partitions; model specification; performance and uncertainty; transparency	Posture/orientation; road-scene coverage; event timeline; detection-referenced stopping
STARD-AI	AI diagnostic-accuracy studies	Index test; reference definition; thresholds; participant/sample flow; uncertainty	Sensor-state degradation; hard road-level negatives; vehicle intervention boundary
CLAIM	Medical-imaging AI	Image/data provenance; preprocessing; architecture; evaluation; failure analysis	Multimodal road sensors; mounting/synchronisation; illumination/weather; scenario matrix
CONSORT-AI	Clinical trials of AI interventions	Intervention description; outputs; human–AI role; harms and failures	Detector-to-actuator timestamps; braking assumptions; measured versus modelled outcomes
NUP-REPORT 1.0	Non-upright pedestrian detection and pre-crash evaluation	Applies the above general reporting principles	Adds posture-specific, sensing, timing, coverage, and vehicle-physics reporting

**Table 6:** Hypothetical worked application of NUP-REPORT.

Checklist Element	Example Entry	Status/Reason
Target/posture	Prone, transverse to path; adult-size heated articulated target	R
Scenario	Night; low-beam headlamps; dry asphalt; 30 km/h; 15 and 30 m	R; rain and occlusion untested
Data independence	60 events; actor/session split; no adjacent-frame leakage	R
Timing	t_obs_ from eligibility gate; t_det_ at first 3-frame criterion; TTFD and d_det_ reported	R
Uncertainty	Event-level bootstrap interval; 2000 resamples	R
Fusion/ablation	Single thermal camera	NA—not multimodal
Vehicle response	No actuator or braking model	NA—no intervention claim

## Data Availability

No participant-level or proprietary data were used. The framework-development ledger, item traceability, pilot-audit codebook, paper list, complete R/NR/NA matrix, item summaries, and figure files are provided with the Supplementary Materials as machine-readable files. All evidence sources are publicly cited.

## Supplementary Materials

The following supporting information can be downloaded at: https://www.mdpi.com/article/10.3390/s26185710/s1, Figure S1: Framework-development and pilot-audit flow; Figure S2: Proposed adoption and validation pathway; Table S1: Reproducible search and selection specification; Table S2: Reconstructed 46-candidate decision ledger; Table S3: Item-to-source traceability matrix; Table S4: Relationship to established AI reporting guidance; Table S5: NUP-REPORT 1.0 checklist with applicability rules; Table S6: Publications included in the purposive pilot feasibility audit; Tables S7a–S7b: Complete pilot coding matrix, Items 1–30; Table S8: Item-level pilot-audit summary; Table S9: Worked application to a hypothetical study. Supplementary Sections S1 and S2 provide the reproducible search protocol, framework-development flow, 46-candidate decision ledger, item-to-source traceability matrix, comparison with AI reporting guidelines, applicability-coded checklist, 20-publication pilot sample and complete coding matrix, item-level summary, worked example, and staged validation pathway. Machine-readable CSV files accompany the supplement.
