# Supplementary material for: NUP-REPORT 1.0: A Proposed Reporting and Benchmarking Framework for Non-Upright Pedestrian Detection and Pre-Crash Safety Evaluation
Source: Sensors (Basel). 2026 Sep 9;26(18):5710. doi: 10.3390/s26185710 (PMC13611086; doi:10.3390/s26185710)
Supplement: Supplementary file 1 [file sensors-26-05710-s001.zip › sensors-4542285-supplementary.pdf]

Supplementary Materials

NUP-REPORT 1.0: A Proposed Reporting and Benchmarking Framework for Non-Upright Pedestrian Detection and Pre-Crash Safety Evaluation

Authors: Nick Barua; Masahito Hitosugi

Department of Legal Medicine, Shiga University of Medical Science, Otsu, Shiga, Japan

Correspondence: s.nick.barua@gmail.com

Contents: S1, evidence-search and source-selection protocol; Figure S1, development and pilot-audit flow; Table S2, 46-candidate decision ledger; Table S3, item-to-source traceability; Table S4, comparison with AI reporting guidelines; Table S5, applicability-coded checklist; Table S6, 20-publication pilot sample; Tables S7a–S7b, full pilot matrix; Table S8, item-level audit summary; Table S9, worked example; and Figure S2, staged validation pathway.

S1. Evidence-search and source-selection protocol

NUP-REPORT was developed through a structured narrative synthesis rather than a systematic review. The update covered database/repository inception through 19 August 2026. The original exploratory search did not preserve a complete export of every hit; consequently, no retrospective PRISMA count or completeness claim is made. The table below provides a reproducible search specification for updating or independently challenging the retained corpus.

Table S1. Reproducible search and selection specification

Element	Prespecified description
**Bibliographic databases**	PubMed/MEDLINE; Scopus; Web of Science; IEEE Xplore; Google Scholar
**Official repositories**	WHO; ISO; Euro NCAP; UNECE; NHTSA; SAE International; EQUATOR Network
**Posture block**	(non-upright OR prone OR supine OR fallen OR lying OR crouched OR seated OR kneeling OR collapse*)
**Road-user/sensing block**	(pedestrian OR vulnerable road user) AND (detect* OR camera OR thermal OR radar OR lidar OR multispectral OR sensor fusion)
**Evaluation block**	(latency OR time-to-detection OR calibration OR uncertainty OR stopping distance OR automatic emergency braking OR reporting guideline)
**Selection**	Title/abstract screening for direct road-transport, sensing, assurance, reporting-method, or safety-protocol relevance; full text reviewed when a source could alter a reporting decision
**Include**	Sources supporting at least one reporting item, definition, applicability rule, benchmark variable, assurance boundary, or development-method decision
**Exclude**	Indoor fall detection without transport relevance; duplicates; marketing material; inaccessible claims; sources that did not alter any reporting decision
**Retained corpus**	45 sources: the 40 sources in the submitted version plus four established AI reporting guidelines and one reporting-guideline development method
**Pilot audit**	20 external publications purposively sampled across classical detection, benchmarks, multispectral methods, multimodal driving data, and fallen-person detection to test checklist breadth and usability. The sample included adjacent literature not designed specifically for non-upright evaluation and was not intended to estimate field-wide reporting prevalence.

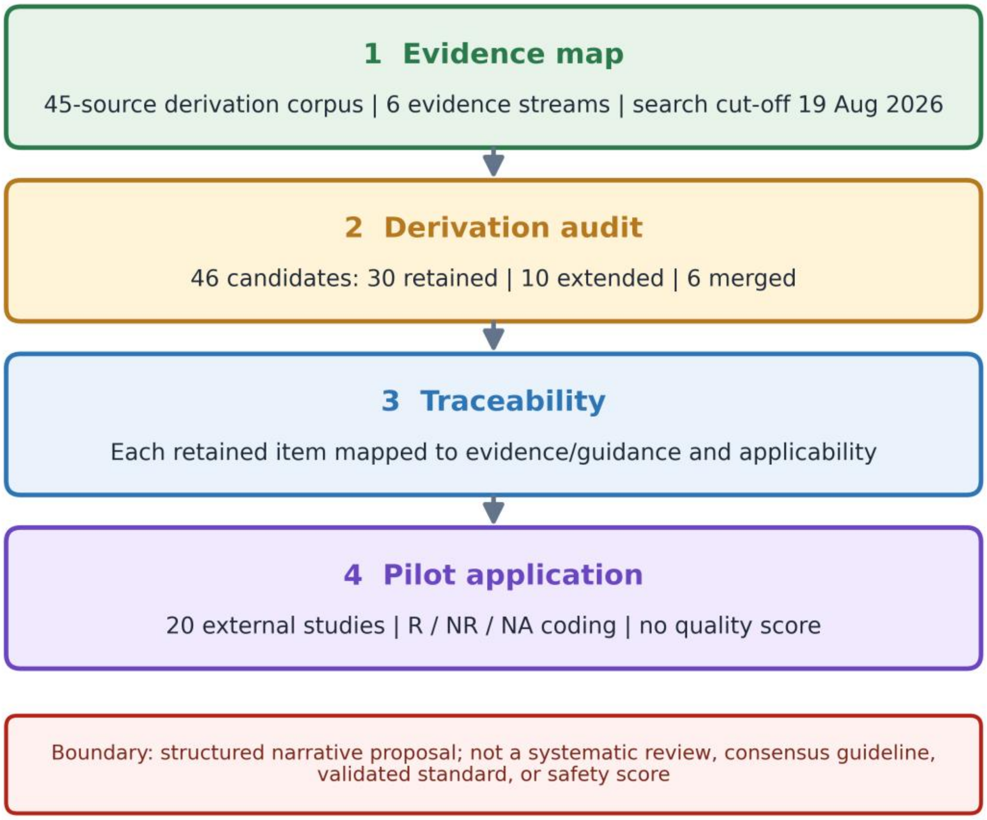

Figure S1. Framework-development and pilot-audit flow. The 46-candidate ledger was reconstructed from dated drafts and source notes; it is not presented as a retrospective PRISMA flow.

Table S2. Reconstructed 46-candidate decision ledger

ID	Domain	Candidate concept	Decision	Rationale/location
1	Target	Define every included posture category operationally	Retained	Checklist Item 1; Core-U
2	Target	State target orientation relative to the vehicle path	Retained	Checklist Item 2; Core-U
3	Target	Distinguish static posture from fall/collapse transition	Retained	Checklist Item 3; Core-U
4	Target	Describe human, mannequin, articulated, or digital target	Retained	Checklist Item 4; Core-U
5	Target	Report body-size category and relevant target properties	Retained	Checklist Item 5; Core-U
6	Target	Include and describe difficult negative objects	Retained	Checklist Item 6; Core-U
7	Scenario	Report ego speed and target motion state	Retained	Checklist Item 7; Core-C
8	Scenario	Report initial range and target lane position	Retained	Checklist Item 8; Core-C
9	Scenario	Report illumination and headlamp state	Retained	Checklist Item 9; Core-U
10	Scenario	Report weather and road-surface condition	Retained	Checklist Item 10; Core-C
11	Scenario	Quantify occlusion and background clutter	Retained	Checklist Item 11; Core-U
12	Data	Report sensor modality, resolution/rate, and mounting	Retained	Checklist Item 12; Core-U
13	Data	Describe calibration and synchronisation	Retained	Checklist Item 13; Core-C
14	Data	Separate real, augmented, and synthetic samples	Retained	Checklist Item 14; Core-U
15	Data	Explain annotation procedure and quality control	Retained	Checklist Item 15; Core-U
16	Data	Use or justify event/actor/location/seed-level partitioning	Retained	Checklist Item 16; Core-U
17	Model	Identify architecture, version, pretraining, and input resolution	Retained	Checklist Item 17; Core-U
18	Model	Report confidence, matching, and persistence thresholds	Retained	Checklist Item 18; Core-U
19	Model	Describe fusion and temporal logic; report missing-modality handling when relevant	Retained	Checklist Item 19; Conditional: fusion/temporal or missing-modality method
20	Model	Provide modality-specific baselines and ablation	Retained	Checklist Item 20; Conditional: multimodal claims
21	Performance	Report counts and posture-specific precision/recall	Retained	Checklist Item 21; Core-U
22	Performance	Report false-negative counts and safety-critical FNR	Retained	Checklist Item 22; Core-U
23	Performance	Report TTFD and distance at first valid detector output	Retained	Checklist Item 23; Conditional: temporal/system evaluation
24	Performance	Report post-detection and observation-to-brake timing	Retained	Checklist Item 24; Conditional: intervention claims
25	Performance	Evaluate confidence calibration	Retained	Checklist Item 25; Conditional: confidence used operationally
26	Performance	Provide uncertainty intervals at the independent-unit level	Retained	Checklist Item 26; Core-U

Table S2. Reconstructed 46-candidate decision ledger (continued)

ID	Domain	Candidate concept	Decision	Rationale/location
27	Performance	Document representative and high-confidence failures	Retained	Checklist Item 27; Core-U
28	Safety	State braking, friction, gradient, and actuator assumptions	Retained	Checklist Item 28; Conditional: vehicle-response modelling
29	Safety	Distinguish measured, simulated, and analytical outcomes	Retained	Checklist Item 29; Core-U
30	Safety	Report stopping margin/residual speed and sensitivity	Retained	Checklist Item 30; Conditional: vehicle-safety claims
31	Target	Detailed limb configuration	Extended	Retained as an extended descriptor under Item 1
32	Target	Head orientation	Extended	Retained as an extended descriptor under Items 1–2
33	Target	Clothing and target emissivity	Extended	Conditional target-fidelity descriptor under Item 5
34	Scenario	Road curvature and gradient geometry	Extended	Conditional scenario descriptor under Items 8 and 28
35	Scenario	Glare intensity	Extended	Conditional illumination descriptor under Item 9
36	Scenario	Precipitation intensity	Extended	Conditional weather descriptor under Item 10
37	Data	Compression and dropped-frame handling	Extended	Conditional data-quality descriptor under Item 12
38	Data	Inter-annotator agreement	Extended	Conditional annotation descriptor under Item 15
39	Model	Parameter count and numerical precision	Extended	Conditional compute descriptor under Item 17
40	Model	Compute-load stress test	Extended	Conditional deployment descriptor under Items 17 and 23
41	Target	Prone and supine as separate checklist items	Merged	Merged into operational posture definition (Item 1)
42	Scenario	Day and night as separate checklist items	Merged	Merged into illumination/headlamp reporting (Item 9)
43	Data	Real and synthetic provenance as separate items	Merged	Merged into sample-source separation (Item 14)
44	Model	Fusion level and missing modality as separate items	Merged	Merged into Item 19
45	Performance	Precision and recall as separate items	Merged	Merged into Item 21
46	Safety	Stopping margin and residual impact speed as separate items	Merged	Merged into Item 30

Table S3. Item-to-source traceability matrix

Item	Domain	Reporting decision	Principal support	Basis
1	Target	Define every included posture category operationally	[2,3,14–17]	Transport-specific proposal informed by evidence
2	Target	State target orientation relative to the vehicle path	[2,3,14–17]	Transport-specific proposal informed by evidence
3	Target	Distinguish static posture from fall/collapse transition	[14–17]	Transport-specific proposal informed by evidence
4	Target	Describe human, mannequin, articulated, or digital target	[14–17,34–36]	Transport-specific proposal informed by evidence
5	Target	Report body-size category and relevant target properties	[8,14–17,34]	Transport-specific proposal informed by evidence
6	Target	Include and describe difficult negative objects	[4–10,14]	Transport-specific proposal informed by evidence
7	Scenario	Report ego speed and target motion state	[2,3,14,24,25,32,33,35–38]	Transport-specific proposal informed by evidence
8	Scenario	Report initial range and target lane position	[2,3,14,24,25,32,33,35–38]	Transport-specific proposal informed by evidence
9	Scenario	Report illumination and headlamp state	[2,3,8,14,21,35,36]	Transport-specific proposal informed by evidence
10	Scenario	Report weather and road-surface condition	[8,21,35,36]	Transport-specific proposal informed by evidence
11	Scenario	Quantify occlusion and background clutter	[4–10,14,21]	Transport-specific proposal informed by evidence
12	Data	Report sensor modality, resolution/rate, and mounting	[8–12,21,34]	Transport-specific proposal informed by evidence
13	Data	Describe calibration and synchronisation	[8,10–12,21,34]	Transport-specific proposal informed by evidence
14	Data	Separate real, augmented, and synthetic samples	[4–14,21,26]	Transport-specific proposal informed by evidence
15	Data	Explain annotation procedure and quality control	[4–14,41–45]	Established guidance plus transport adaptation
16	Data	Use or justify event/actor/location/seed-level partitioning	[4–14,23–29,41–45]	Established guidance plus transport adaptation
17	Model	Identify architecture, version, pretraining, and input resolution	[4,5,14,21,41–44,46–52]	Established guidance plus transport adaptation
18	Model	Report confidence, matching, and persistence thresholds	[14,18,41–44,46–52]	Established guidance plus transport adaptation
19	Model	Describe fusion and temporal logic; report missing-modality handling when relevant	[15–17,21,41–44,47–51]	Transport-specific proposal informed by evidence
20	Model	Provide modality-specific baselines and ablation	[15–17,21,41–44,47–51]	Transport-specific proposal informed by evidence
21	Performance	Report counts and posture-specific precision/recall	[4–14,41–45]	Established guidance plus transport adaptation
22	Performance	Report false-negative counts and safety-critical FNR	[2,3,14–17,41–45]	Transport-specific proposal informed by evidence
23	Performance	Report TTFD and distance at first valid detector output	[14–17,24,25,32–39]	Transport-specific proposal informed by evidence
24	Performance	Report post-detection and observation-to-brake timing	[15–17,30–39]	Transport-specific proposal informed by evidence
25	Performance	Evaluate confidence calibration	[18–20,22,23,27,41–45]	Established guidance plus transport adaptation
26	Performance	Provide uncertainty intervals at the independent-unit level	[18,19,22,23,27,41–45]	Established guidance plus transport adaptation

Table S3. Item-to-source traceability matrix (continued)

Item	Domain	Reporting decision	Principal support	Basis
27	Performance	Document representative and high-confidence failures	[14–23,41–45]	Established guidance plus transport adaptation
28	Safety	State braking, friction, gradient, and actuator assumptions	[15–17,24,25,28–38]	Transport-specific proposal informed by evidence
29	Safety	Distinguish measured, simulated, and analytical outcomes	[15–17,24,25,28–38]	Transport-specific proposal informed by evidence
30	Safety	Report stopping margin/residual speed and sensitivity	[15–17,24,25,28–38]	Transport-specific proposal informed by evidence

Table S4. Relationship to established AI reporting guidance

Framework	Scope	General element informing NUP-REPORT	NUP-REPORT addition/boundary
TRIPOD+AI [41]	Prediction models	Data partitions, model specification, performance, uncertainty, transparency	Adds posture, road-scene, sensing, timing, and physical intervention reporting
STARD-AI [42]	AI diagnostic accuracy	Reference definition, thresholds, sample flow, uncertainty	Adapts the logic to sensor targets, event timelines, and intervention boundaries
CLAIM [43]	Medical imaging AI	Data provenance, preprocessing, architecture, evaluation, failures	Adds multimodal road sensors, environment, mounting, synchronisation, and coverage
CONSORT-AI [44]	AI clinical trials	Intervention description, output, human–AI role, harms	Adds detector-to-actuator timestamps and vehicle-physics assumptions
Guideline-development method [45]	Reporting-guideline development	Transparency, evidence base, stakeholder involvement, pilot testing, updating	NUP-REPORT explicitly identifies incomplete consensus and validation steps

Table S5. NUP-REPORT 1.0 checklist with applicability rules. The 23 core items comprise 19 universal core (Core-U) items and four component-contingent core (Core-C) items (7, 8, 10, and 13); seven items are conditional.

No.	Domain	Reporting item	Applicability	Reported/location
1	Target	Define every included posture category operationally	Core-U	
2	Target	State target orientation relative to the vehicle path	Core-U	
3	Target	Distinguish static posture from fall/collapse transition	Core-U	
4	Target	Describe human, mannequin, articulated, or digital target	Core-U	
5	Target	Report body-size category and relevant target properties	Core-U	
6	Target	Include and describe difficult negative objects	Core-U	
7	Scenario	Report ego speed and target motion state	Core-C	
8	Scenario	Report initial range and target lane position	Core-C	
9	Scenario	Report illumination and headlamp state	Core-U	
10	Scenario	Report weather and road-surface condition	Core-C	
11	Scenario	Quantify occlusion and background clutter	Core-U	
12	Data	Report sensor modality, resolution/rate, and mounting	Core-U	
13	Data	Describe calibration and synchronisation	Core-C	
14	Data	Separate real, augmented, and synthetic samples	Core-U	
15	Data	Explain annotation procedure and quality control	Core-U	
16	Data	Use or justify event/actor/location/seed-level partitioning	Core-U	
17	Model	Identify architecture, version, pretraining, and input resolution	Core-U	
18	Model	Report confidence, matching, and persistence thresholds	Core-U	
19	Model	Describe fusion and temporal logic; report missing-modality handling when relevant	Conditional	
20	Model	Provide modality-specific baselines and ablation	Conditional	
21	Performance	Report counts and posture-specific precision/recall	Core-U	
22	Performance	Report false-negative counts and safety-critical FNR	Core-U	
23	Performance	Report TTFD and distance at first valid detector output	Conditional	
24	Performance	Report post-detection and observation-to-brake timing	Conditional	
25	Performance	Evaluate confidence calibration	Conditional	
26	Performance	Provide uncertainty intervals at the independent-unit level	Core-U	

Table S5. NUP-REPORT 1.0 checklist with applicability rules (continued)

No.	Domain	Reporting item	Applicability	Reported/location
27	Performance	Document representative and high-confidence failures	Core-U	
28	Safety	State braking, friction, gradient, and actuator assumptions	Conditional	
29	Safety	Distinguish measured, simulated, and analytical outcomes	Core-U	
30	Safety	Report stopping margin/residual speed and sensitivity	Conditional	

Table S6. Publications included in the purposive pilot feasibility audit. The sample intentionally included adjacent pedestrian-detection and benchmark literature; only P20 directly evaluated fallen-person detection.

ID	Publication	Year	Study type	Reference
P01	Dalal and Triggs	2005	HOG human detection	[5]
P02	Dollár et al.	2012	Pedestrian-detection evaluation	[4]
P03	Geiger et al.	2012	KITTI	[10]
P04	Hwang et al.	2015	KAIST multispectral benchmark	[8]
P05	Zhang et al.	2016	Pedestrian-detection benchmark analysis	[46]
P06	Cordts et al.	2016	Cityscapes	[9]
P07	Liu et al.	2016	Multispectral pedestrian detection	[47]
P08	Zhang et al.	2017	CityPersons	[6]
P09	Xu et al.	2017	Cross-modal pedestrian detection	[48]
P10	König et al.	2017	Multispectral region proposal network	[49]
P11	Rasouli et al.	2017	JAAD	[13]
P12	Braun et al.	2019	EuroCity Persons	[7]
P13	Zhang et al.	2019	Weakly aligned cross-modal detection	[50]
P14	Rasouli et al.	2019	PIE	[26]
P15	Caesar et al.	2020	nuScenes	[11]
P16	Sun et al.	2020	Waymo Open Dataset	[12]
P17	Bijelic et al.	2020	Adverse-weather multimodal fusion	[21]
P18	Wolpert et al.	2020	Small-scale multispectral detection	[51]
P19	Hasan et al.	2021	Generalizable pedestrian detection	[52]
P20	Lee et al.	2023	Fallen-person detection	[14]

Table S7a. Complete pilot coding matrix, Items 1–15

Paper	1	2	3	4	5	6	7	8	9	10	11	12	13	14	15
P01	R	NR	NR	R	NR	R	NA	NA	NR	NA	NR	R	NA	R	R
P02	R	NR	NR	R	R	R	NA	NR	NR	NR	R	R	NR	R	R
P03	R	NR	NR	R	R	NR	NR	R	R	R	R	R	R	R	R
P04	R	NR	NR	R	R	NR	NR	NR	R	NR	R	R	R	R	R
P05	R	NR	NR	R	R	R	NA	NR	NR	NR	R	R	NR	R	R
P06	R	NR	NR	R	R	R	NR	NR	R	R	R	R	R	R	R
P07	R	NR	NR	R	R	NR	NA	NA	R	NR	R	R	R	R	R
P08	R	NR	NR	R	R	R	NR	NR	R	NR	R	R	NR	R	R
P09	R	NR	NR	R	NR	NR	NA	NA	R	NR	R	R	R	R	R
P10	R	NR	NR	R	R	NR	NA	NA	R	NR	R	R	R	R	R
P11	R	R	R	R	R	R	R	NR	R	R	R	R	NR	R	R
P12	R	NR	NR	R	R	R	NR	NR	R	R	R	R	R	R	R
P13	R	NR	NR	R	R	NR	NA	NA	R	NR	R	R	R	R	R
P14	R	R	R	R	R	R	R	R	R	R	R	R	NR	R	R
P15	R	R	R	R	R	R	R	R	R	R	R	R	R	R	R
P16	R	R	R	R	R	R	R	R	R	R	R	R	R	R	R
P17	R	NR	NR	R	R	R	NR	R	R	R	R	R	R	R	R
P18	R	NR	NR	R	R	NR	NA	NA	R	NR	R	R	R	R	R
P19	R	NR	NR	R	R	R	NA	NR	R	R	R	R	NA	R	R
P20	R	R	R	R	R	R	NR	NR	R	R	R	R	NR	R	R

Coding: R, explicitly reported; NR, applicable but not reported; NA, prespecified applicability trigger absent. Core-U items were coded R or NR; Core-C items (7, 8, 10, and 13) and conditional items could be coded NA only when their trigger was genuinely absent. Because the purposive audit included adjacent literature not designed specifically for non-upright evaluation, applicable denominators vary. No total score was calculated.

Table S7b. Complete pilot coding matrix, Items 16–30

Paper	16	17	18	19	20	21	22	23	24	25	26	27	28	29	30
P01	R	R	R	NA	NA	NR	R	NA	NA	NR	NR	R	NA	R	NA
P02	R	R	R	NA	NA	NR	R	NA	NA	NR	NR	R	NA	R	NA
P03	R	R	R	NR	R	NR	R	NR	NA	NR	NR	NR	NA	R	NA
P04	R	R	R	NR	R	NR	R	NR	NA	NR	NR	NR	NA	R	NA
P05	R	R	R	NA	NA	NR	R	NA	NA	NR	NR	R	NA	R	NA
P06	R	R	R	NA	NA	NR	R	NR	NA	NA	NR	NR	NA	R	NA
P07	R	R	R	NR	R	NR	R	NR	NA	NR	NR	NR	NA	R	NA
P08	R	R	R	NA	NA	NR	R	NR	NA	NR	NR	R	NA	R	NA
P09	R	R	R	NR	R	NR	R	NR	NA	NR	NR	NR	NA	R	NA
P10	R	R	R	NR	R	NR	R	NR	NA	NR	NR	NR	NA	R	NA
P11	R	R	R	R	NA	NR	R	NR	NA	NA	NR	R	NA	R	NA
P12	R	R	R	NA	NA	NR	R	NR	NA	NR	NR	R	NA	R	NA
P13	R	R	R	NR	R	NR	R	NR	NA	NR	NR	R	NA	R	NA
P14	R	R	R	R	NA	NR	R	NR	NA	NA	NR	R	NA	R	NA
P15	R	R	R	NR	R	NR	R	NR	NA	NR	NR	NR	NA	R	NA
P16	R	R	R	NR	R	NR	R	NR	NA	NR	NR	NR	NA	R	NA
P17	R	R	R	R	R	NR	R	NR	NA	NR	NR	R	NA	R	NA
P18	R	R	R	NR	R	NR	R	NR	NA	NR	NR	NR	NA	R	NA
P19	R	R	R	NA	NA	NR	R	NR	NA	NR	NR	R	NA	R	NA
P20	R	R	R	NA	NA	R	R	NR	NA	NR	NR	R	NA	R	NA

Coding: R, explicitly reported; NR, applicable but not reported; NA, prespecified applicability trigger absent. Core-U items were coded R or NR; Core-C items (7, 8, 10, and 13) and conditional items could be coded NA only when their trigger was genuinely absent. Because the purposive audit included adjacent literature not designed specifically for non-upright evaluation, applicable denominators vary. No total score was calculated.

Table S8. Item-level pilot-audit summary. Applicable denominators reflect the prespecified Core-C and conditional triggers; percentages describe this purposive sample only.

Item	Domain	Short label	Applicability	R	Applicable	% R
1	Target	Operational target/posture definition	Core-U	20	20	100.0
2	Target	Orientation relative to vehicle path	Core-U	5	20	25.0
3	Target	Static posture versus transition	Core-U	5	20	25.0
4	Target	Human/mannequin/digital target type	Core-U	20	20	100.0
5	Target	Body size and relevant target properties	Core-U	18	20	90.0
6	Target	Difficult negative objects	Core-U	13	20	65.0
7	Scenario	Ego speed and target motion state	Core-C	4	11	36.4
8	Scenario	Initial range and lane position	Core-C	5	14	35.7
9	Scenario	Illumination and headlamp state	Core-U	17	20	85.0
10	Scenario	Weather and road-surface condition	Core-C	10	19	52.6
11	Scenario	Occlusion and background clutter	Core-U	19	20	95.0
12	Data	Sensor modality, resolution/rate and mounting	Core-U	20	20	100.0
13	Data	Calibration and synchronization	Core-C	12	18	66.7
14	Data	Real, augmented and synthetic samples separated	Core-U	20	20	100.0
15	Data	Annotation procedure and quality control	Core-U	20	20	100.0
16	Data	Event/actor/location/seed-level partitioning	Core-U	20	20	100.0
17	Model	Architecture/version/pretraining/input resolution	Core-U	20	20	100.0
18	Model	Confidence, matching and persistence thresholds	Core-U	20	20	100.0
19	Model	Fusion/temporal logic; missing-modality handling when applicable	Conditional	3	12	25.0
20	Model	Modality baselines and ablation	Conditional	10	10	100.0
21	Performance	Counts and posture-specific precision/recall	Core-U	1	20	5.0
22	Performance	False-negative counts or safety-critical FNR	Core-U	20	20	100.0
23	Performance	TTFD and distance at first detector output	Conditional	0	17	0.0
24	Performance	Post-detection and observation-to-brake timing	Conditional	0	0	NA
25	Performance	Confidence calibration	Conditional	0	17	0.0

Table S8. Item-level pilot-audit summary (continued)

Item	Domain	Short label	Applicability	R	Applicable	% R
26	Performance	Uncertainty intervals at independent-unit level	Core-U	0	20	0.0
27	Performance	Representative and high-confidence failures	Core-U	11	20	55.0
28	Safety	Braking/friction/gradient/actuator assumptions	Conditional	0	0	NA
29	Safety	Measured/simulated/analytical status	Core-U	20	20	100.0
30	Safety	Stopping margin/residual speed and sensitivity	Conditional	0	0	NA

Table S9. Worked application to a hypothetical study

Domain/item	Hypothetical thermal-camera study entry	Code
**1–5 Target**	Prone, transverse, stationary adult-size heated articulated target; geometric and thermal properties stated	R
**6 Difficult negatives**	Blankets, bags, shadows, and road debris included	R
**7–11 Scenario**	30 km/h; 15/30 m; lane centre; darkness/low beam; dry road; no occlusion	R; rain/occlusion untested
**12–16 Data**	Thermal camera specifications and mounting; calibration; 60 event-independent sequences; annotations double-checked	R
**17–19 Model**	Architecture, input, threshold, three-frame detector criterion; single modality	R
**20 Multimodal ablation**	No fusion claim	NA
**21–23 Performance/timing**	Event counts, precision/recall, misses, t_obs/t_det, TTFD, and d_det with event-level intervals	R
**24 Post-detection timing**	No controller or actuator evaluated	NA
**25 Calibration**	Confidence used only to define detection; reliability diagram and ECE supplied	R
**26–27 Uncertainty/failures**	Event bootstrap; representative and high-confidence failures shown	R
**28–30 Vehicle safety**	No braking, stopping, or impact claim	NA

S2. Interpretation of the worked example

The example demonstrates that Core-C and conditional items are not treated as missing when their prespecified trigger is absent. It also shows that an explicit detection event requires a declared persistence rule: if t_det is the first detector output satisfying a three-frame detector criterion, those three frames are part of TTFD; any additional control-side confirmation begins after t_det. If distance is measured at t_det, only post-detection motion is used in stopping calculations.

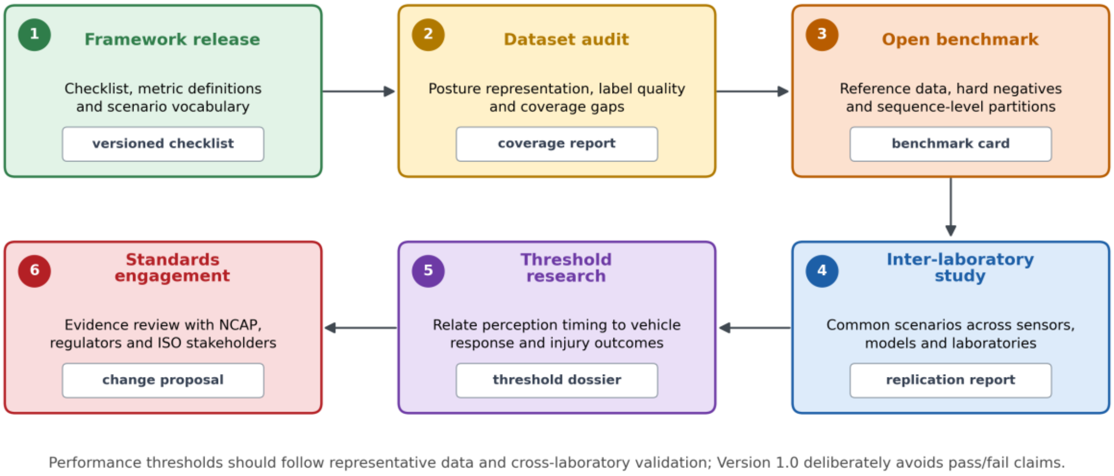

Figure S2. Proposed adoption and validation pathway. Performance thresholds and protocol changes should follow representative data, independent coding, stakeholder consensus, inter-laboratory evidence, and formal review.
